# Draft Genome Sequence of Streptomyces sp. Strain CT34, Isolated from a Ghanaian Soil Sample

**DOI:** 10.1128/genomeA.01508-14

**Published:** 2015-02-05

**Authors:** Yin Zhai, Bin Cheng, Juan Hu, Kwaku Kyeremeh, Xiaoling Wang, Marcel Jaspars, Hai Deng, Zi-Xin Deng, Kui Hong

**Affiliations:** a Key Laboratory of Combinatorial Biosynthesis and Drug Discovery, Ministry of Education, School of Pharmaceutical Sciences, Wuhan University, Wuhan, China; b Beijing Genomics Institution, Shenzhen, China; c Department of Chemistry, University of Ghana, Accra, Ghana; d Marine Biodiscovery Centre, Department of Chemistry, University of Aberdeen, Aberdeen, United Kingdom

## Abstract

Presented here is a draft genome sequence of Streptomyces sp. strain CT34, which produces a novel ribosomally synthesized and posttranslationally modified peptide (RiPP). Analysis of the deduced open reading frame set identified the putative RiPP biosynthesis gene cluster, as well as other secondary metabolite gene clusters.

## GENOME ANNOUNCEMENT

The newly emerged ribosomally synthesized and post-translationally modified peptides (RiPPs) form a very large group of important natural products with vast structure diversity (1). A new RiPP with a molecular weight of 3.6 kDa was isolated from Ghanaian Streptomyces sp. strain CT34. The structure of the peptide is quite different from the current ribosomal peptides, and it appears to fall into the linaridin class (2).

Genomic DNA of strain CT34 was extracted via the method of Pospiech and Neumann (3) and submitted for whole-genome sequencing by Beijing Genomics Institution (BGI). Two types of 500-bp and 6-Kb sequence libraries were prepared and sequenced by the Illumina HiSeq2000. A total of 24,826,204 raw reads were obtained with two libraries of 904 Mbp and 451 Mbp after deletion of the low-quality and adapter-containing reads (4). The resulting reads were assembled into 16 scaffolds and 310 contigs using SOAPdenovo (5). The total length of the assembly was 8,066,430 bp, with a genome coverage of 99.85% and an average GC content of approximately 71.39%.

Glimmer software (6, 7) was used to predict the genes of Streptomyces sp. CT34. The genome possesses 7,781 genes, with an average length of 875 bp. The predicted total gene length was 6,809,991 bp, which makes up 84.42% of the genome. Based on the analysis of the antiSMASH (8) program, 30 gene clusters were revealed for secondary metabolites biosynthesis, including 3 siderophores, 4 terpenes, 1 mixed nonribosomal peptide synthetase (NRPS)/polyketide synthase (T1-PKS), 1 mixed oligosaccharide/terpene, 1mixed PKS (T4-PKS)/PKS (T1-PKS), 1 mixed lantipeptide/PKS (T1-PKS), 3 PKSs (2 T2-PKSs, 1 T3-PKS), 2 lantipeptides, 3 NRPSs, 3 bacteriocins, 3 butyrolactones, 1 ectoine, and 4 unspecified clusters. A putative gene cluster related to the biosynthesis of the new linaridin RiPP was revealed with a length of 12,108 bp, including 10 open reading frames (ORFs) of catalytic and auxiliary functions and 1 ORF encoding the prepeptide. Further analysis of the novel peptide is under way.

### Nucleotide sequence accession numbers.

This whole-genome shotgun project has been deposited at DDBJ/EMBL/GenBank under the accession number JSFP00000000. The version described in this paper is version JSFP01000000.
